# The Use of Targeted Marker Subsets to Account for Population Structure and Relatedness in Genome-Wide Association Studies of Maize (*Zea mays* L.)

**DOI:** 10.1534/g3.116.029090

**Published:** 2016-05-26

**Authors:** Angela H. Chen, Alexander E. Lipka

**Affiliations:** * Department of Statistics, University of Illinois at Urbana-Champaign, Illinois 61801; † Department of Crop Sciences, University of Illinois at Urbana-Champaign, Illinois 61801

**Keywords:** GWAS, mixed model, linkage disequilibrium, maize, marker subsets

## Abstract

A typical plant genome-wide association study (GWAS) uses a mixed linear model (MLM) that includes a trait as the response variable, a marker as an explanatory variable, and fixed and random effect covariates accounting for population structure and relatedness. Although effective in controlling for false positive signals, this model typically fails to detect signals that are correlated with population structure or are located in high linkage disequilibrium (LD) genomic regions. This result likely arises from each tested marker being used to estimate population structure and relatedness. Previous work has demonstrated that it is possible to increase the power of the MLM by estimating relatedness (*i.e.*, kinship) with markers that are not located on the chromosome where the tested marker resides. To quantify the amount of additional significant signals one can expect using this so-called K_chr model, we reanalyzed Mendelian, polygenic, and complex traits in two maize (*Zea mays* L.) diversity panels that have been previously assessed using the traditional MLM. We demonstrated that the K_chr model could find more significant associations, especially in high LD regions. This finding is underscored by our identification of novel genomic signals proximal to the tocochromanol biosynthetic pathway gene *ZmVTE1* that are associated with a ratio of tocotrienols. We conclude that the K_chr model can detect more intricate sources of allelic variation underlying agronomically important traits, and should therefore become more widely used for GWAS. To facilitate the implementation of the K_chr model, we provide code written in the R programming language.

The evaluation of associations between a set of genomic markers and a trait of interest makes it possible to obtain biological insight into the relationship between genetic and phenotypic variation. This can potentially culminate in the identification of specific genes associated with the trait and a rigorous assessment of the ability of the markers to collectively predict trait values (reviewed in Lipka *et al.* 2015). Given recent advances in genotyping technologies and corresponding cost reductions, analyses that utilize genome-wide marker sets to study the genetic components underlying phenotypic variation are becoming increasingly commonplace (Zhu *et al.* 2008; Daetwyler *et al.* 2013; Flint and Eskin 2012). One such analysis is the genome-wide association study (GWAS), where markers spanning an entire genome are tested for associations with a group of traits in a panel consisting of a diverse set of individuals (Myles *et al.* 2009). Because a typical diversity panel captures a substantial amount of allelic diversity and historical recombination events, it is assumed that a marker identified in a GWAS as significantly associated with a trait is in strong linkage disequilibrium (LD) with one or more causal genomic variants (Platt *et al.* 2010).

An important drawback of the GWAS is that false positive marker-trait associations due to population structure and familial relatedness could arise if unaccounted for (Yu *et al.* 2006; Lipka *et al.* 2015; Zhang *et al.* 2010). The ability to adjust for such sources of false positives in a computationally efficient manner has been an active area of research (Kang *et al.* 2008, 2010; Zhou and Stephens 2012); consequently, a typical GWAS in plants will employ statistical approaches that take population structure and familial relatedness into account (Lipka *et al.* 2015).

Of all the state of the art statistical approaches that have been developed to control for false positive marker-trait associations, the unified mixed linear model (MLM; Yu *et al.* 2006) is arguably the best suited for GWAS data sets. In addition to including a tested marker as a fixed effect, this model includes fixed effect covariates that account for population structure and a random polygenic effect to control for relatedness among the individuals. To ensure that these additional terms adequately adjust for false positive signals, genome-wide marker sets are usually used to obtain the fixed effect covariates (called Q), as well as a kinship matrix (K) that estimates the variance-covariance among the individuals (*i.e.*, the variance-covariance of the random polygenic effect). In general, the unified MLM has been successful in identifying marker-trait associations with moderate to large effect sizes (Lipka *et al.* 2015), with some notable examples in maize including signals proximal to candidate genes for flowering time (Romay *et al.* 2013) and tocochromanol and carotenoid biosynthesis (Lipka *et al.* 2013; Owens *et al.* 2014). Nevertheless, one impediment of this model is that it has been generally unable to detect small effect loci that underlie complex trait variation (as described in Atwell *et al.* 2010). Thus, there remains a critical need to modify the traditional unified MLM so that it has sufficient statistical power to detect these weak signals, while still adequately controlling for false positives.

Recent studies have identified two particular situations in which the unified MLM overcorrects for false positive signals. The first situation arises when a trait under study is correlated with population structure (Larsson *et al.* 2013). For such a trait, markers in strong LD with a putatively causal locus will likely not be detected using the unified MLM because they are strongly correlated with the fixed effects Q. The second situation occurs when a genomic signal is located in a region of high LD. It has been shown that a failure to detect such signals arises because the common procedures used to calculate the kinship matrix gives more weight to genomic regions containing markers in strong LD (Rincent *et al.* 2014). In both of these situations, failure to detect such signals is likely attributable to the fact that each marker being tested for associations is typically also used to estimate population structure and relatedness. This failure could also occur if a GWAS is conducted on an independent marker set that captures the same degree of population structure and relatedness as the original marker set used to calculate Q and K. To account for these deficiencies, it has therefore been suggested that only certain subsets of genomic markers be used to account for these sources of false positives (Listgarten *et al.* 2012; Bernardo 2013). In particular, Rincent *et al.* (2014) explored the statistical power that was gained by using kinship matrices that were calculated with markers that were not in LD with a given marker being tested. By directly calculating power and evaluating three maize diversity panels, it was concluded that using kinship matrices specific to each chromosome in the unified MLM could result in greater statistical power to detect associations (an approach called the “K_Chr” model). As such, this approach has great potential to enable the unified MLM to identify a greater amount of statistically significant marker-trait associations, while simultaneously controlling for false positives.

Given the increased availability of high density marker sets obtained from the latest sequencing technologies, the GWAS is likely to continue to play a predominant role in unraveling the genetic architecture underlying important traits in a wide variety of species (Korte and Farlow 2013). To facilitate an accurate dissection of genomic signals, it is essential to use a statistical approach that maximizes the power for detecting associations. Therefore, the objective of this study was to evaluate the ability of the K_chr model, proposed by Rincent *et al.* (2014), to provide further insight into the genomic signals that underlie traits in two maize diversity panels that have been previously analyzed using the traditional unified MLM. We hypothesized that the K_chr model would provide the greatest enhancement in genomic resolution for signals located in high LD regions.

## Materials and Methods

### Sources of phenotypic and genotypic data

#### Goodman diversity panel:

In this study, we analyzed publicly available phenotypic and genotypic data from two maize diversity panels. The first panel was the Goodman diversity panel (described in Flint-Garcia *et al.* 2005), which consists of 281 diverse maize lines. To assess the performance of the K_chr model under different genetic architectures, we considered three classes of phenotypes that have been previously assessed via GWAS in this panel (Table 1). The first class of phenotypes included 15 carotenoid compounds, sums, ratios, and proportions that were obtained on a subset of 201 maize lines with kernel color ranging from light yellow to dark orange (originally published in Owens *et al.* 2014). The relatively small number of genes underlying these traits makes it possible for maize breeders to substantially increase essential nutrients, including provitamin A, in maize kernels by selecting on targeted genomic regions containing carotenoid biosynthetic and related genes. As such, our analysis of carotenoids provided an essential counterpoint to the other polygenic and complex traits considered for this study.

**Table 1:** Summary information for three classes of traits that were analyzed in the Goodman association panel described in Flint-Garcia *et al.* (2005)

Trait Class	No. Traits Analyzed	Sample Size	No. Markers for GWAS	Data Source
Carotenoid	15	201	291,939	Owens *et al.* 2014
Tocochromanol	20	252	293,863	Lipka *et al.* 2013
Flowering time	3	278	299,253	www.maizegenetics.net/tassel

GWAS, genome-wide association study.

The second set of phenotypes included 20 tocochromanol compounds, sums, ratios, and proportions that were published in Lipka *et al.* (2013). Similar to carotenoids, tocochromanol compounds have a tractable genetic architecture in maize, which could allow breeders to increase vitamin E and antioxidant levels in maize grain by selecting on a small number of genes in the tocochromanol biosynthetic and precursor pathways. However, previous QTL analyses in maize grain suggest that tocochromanols are controlled by more genes relative to carotenoids (Wong *et al.* 2003; Chander *et al.* 2008; Kandianis *et al.* 2013), thus making tocochromanols an ideal set of polygenic traits to evaluate with the K_chr model.

The final set of phenotypes we evaluated included three phenotypes related to flowering time (*i.e.*, days to pollen, ear height, and ear diameter). Because of their complex genetic architecture and importance for breeding, flowering time-related phenotypes have been extensively studied in maize association studies (*e.g.*, Flint-Garcia *et al.* 2005; Salvi *et al.* 2007; Buckler *et al.* 2009; Thornsberry *et al.* 2001; Larsson *et al.* 2013; Romay *et al.* 2013; Peiffer *et al.* 2014), yet the characterization of the vast majority of the loci underlying their genetic variability has remained elusive. Therefore, it will be critical to determine the extent to which the K_chr model can identify loci associated with flowering time, especially those located in regions of high LD.

The genome-wide SNPs used in this study have been previously described (Lipka *et al.* 2013; Larsson *et al.* 2013; Owens *et al.* 2014). Briefly, these markers were obtained from the MaizeSNP50 BeadChip (Cook *et al.* 2012; available at http://cbsusrv04.tc.cornell.edu/users/panzea/download.aspx?filegroupid=7), the genotyping-by-sequencing (GBS) protocol (Elshire *et al.* 2011; available at http://cbsusrv04.tc.cornell.edu/users/panzea/download.aspx?filegroupid=5), and several other SNP genotyping assays (Yu *et al.* 2006; McMullen *et al.* 2009). Summary statistics for these three marker sets are included in Supplemental Material, Table S1. Upon removal of SNPs that either i) exhibited minor allele frequency (MAF) of less than 0.05, ii) had low quality scores, or iii) were not anchored to the B73 RefGen_v2 genome assembly (removal of such SNPs is critical for the K_chr model), between 291,939–299,253 SNPs were available for the three phenotypic classes (Table 1). To enable direct comparisons with the results from previous studies, all markers were conservatively imputed with the major allele prior to the GWAS.

#### USDA-ARS North Central Regional Plant Introduction Station (NCRPIS) panel:

The second maize diversity panel we analyzed was the 2815-member NCRPIS panel (described in Romay *et al.* 2013). Consisting of ∼10 times as many individuals as the Goodman diversity panel, the ability of the NCRPIS panel to detect loci associated with traits including flowering time and plant height has been demonstrated (Romay *et al.* 2013; Peiffer *et al.* 2014). We analyzed a total of five publicly available phenotypes in this panel (Table 2). The first of these was a Mendelian trait, namely sweet corn *vs.* starchy corn. A GWAS of this trait conducted in Romay *et al.* (2013) identified peak associations for sweet corn *vs.* starchy corn in a chromosome 4 region containing the kernel starch biosynthesis gene *Su1*. Because the selection pressure of sweet *vs.* starchy corn resulted in high LD in this genomic region (Romay *et al.* 2013), our analysis of this trait enabled a direct comparison of the ability of the K_chr model and the traditional unified MLM to detect associations under elevated LD. The remaining four phenotypes analyzed were related to flowering time and plant height (*i.e.*, days to silking, days to anthesis, plant height, and ear height). When analyzed using the traditional unified MLM in Romay *et al.* (2013) and Peiffer *et al.* (2014), it was demonstrated that the sample size of the NCRPIS panel was sufficient to identify genomic signals associated with these complex traits. Thus, our reanalysis of these four phenotypes made it possible to assess the capability of the K_chr model to provide further elucidation into the genomic sources of complex trait variation.

**Table 2:** Summary information for the traits that were analyzed in the North Central Regional Plant Introduction Station maize association panel described in Romay *et al.* (2013)

Trait	Sample Size	No. Markers for GWAS	Data Source
Sweet *vs.* starchy	2631	387,612	Romay *et al.* 2013
Days to silking	2279	391,060	Romay *et al.* 2013
Days to anthesis*^a^*	2293	391,044	Peiffer *et al.* 2014
Plant height*^a^*	2293	391,044	Peiffer *et al.* 2014
Ear height*^a^*	2293	391,044	Peiffer *et al.* 2014

GWAS, genome-wide association study.

a Both best linear unbiased predictors and best linear unbiased estimators of these three traits are available in the supplement of Peiffer *et al.* 2014. We used best linear unbiased estimators for this analysis.

The genome-wide SNP set used to analyze the NCRPIS panel has been previously described (Romay *et al.* 2013), and summary statistics are included in Table S1. Briefly, these markers were obtained using the GBS protocol (Elshire *et al.* 2011) and are publicly available at http://cbsusrv04.tc.cornell.edu/users/panzea/download.aspx?filegroupid=6. All SNPs with MAF < 0.05 were removed, resulting in between 387,612–391,060 markers that were available for the GWAS. To be consistent with the procedures conducted in Romay *et al.* (2013) and Peiffer *et al.* (2014), all markers were imputed with the heterozygote prior to conducting the GWAS.

### Evaluation of K_chr in GWAS

The GWAS of the carotenoid, tocochromanol, flowering time, and plant height traits was conducted using procedures that are similar to those described in Larsson *et al.* (2013), Lipka *et al.* (2013), Romay *et al.* (2013), Peiffer *et al.* (2014), and Owens *et al.* (2014). The only major difference was that the K_chr model (described in Rincent *et al.* 2014) was used in place of the traditional unified MLM. Briefly, the K_chr model is stated as follows:

Y=Qv+Sα+Zu+ε, (1)

where Y is a vector of observed trait values among *n* individuals, v is a vector of population substructure effects, Q is a matrix of covariates relating v to Y, α is a vector of marker effects, S is an incidence matrix relating α to Y, u is a vector of polygenic effects accounting for relatedness among the individuals, Z is an incidence matrix relating u to Y, and ε is a vector of residual effects. u is a random effect with variance $Var\left(u\right)=2KV_{G}$, while ε is a random effect with variance $Var\left(\varepsilon \right)=IV_{E}$, where K is a kinship matrix quantifying the degree of relatedness between the individuals, $V_{G}$ is the genetic variance, I is the identity matrix, and $V_{E}$ is the residual variance. In the traditional unified MLM, marker sets that capture genome-wide variability are used to calculate both Q and K (Yu *et al.* 2006). In contrast, the K_chr model pioneered by Rincent *et al.* (2014) calculates a separate kinship matrix for each chromosome. That is, for a given chromosome, the kinship matrix is calculated using all markers throughout the genome except for those that reside on that chromosome. In this work, we also use a similar approach to obtain separate Q matrices for each chromosome.

#### Statistical model specific to the Goodman diversity panel:

The K_chr model, as described in Equation 1, was used for the GWAS in both diversity panels. In the Goodman diversity panel, all 37,824 nonindustry SNPs from the Illumina MaizeSNP50 BeadChip that were anchored to a unique B73 RefGen_v2 position except those located on the chromosome under consideration were used to calculate a Loiselle kinship matrix (Loiselle *et al.* 1995) accounting for relatedness and to obtain principal components (PCs; Price *et al.* 2006) accounting for population structure. The unified MLM (Yu *et al.* 2006) with population parameters previously determined (P3D; Zhang *et al.* 2010) was then used in the Genome Association and Prediction Integrated Tool (GAPIT) package (Lipka *et al.* 2012) in the R programming language (R Core Team 2015), to evaluate the association between each marker and trait.

For each trait, the GWAS statistical model was optimized and marker-trait associations were evaluated using the same procedures described in Lipka *et al.* (2013). Briefly, the Bayesian information criterion (BIC; Schwarz 1978) was implemented to determine the most favorable number of PCs to include in the model as covariates. For each evaluated trait, the optimal number of PCs was zero, suggesting that either these evaluated traits were not strongly correlated with population structure or that the kinship matrix is accounting for population structure in addition to familial relatedness. The amount of phenotypic variation explained by the model was evaluated using a likelihood-ratio-based *R^2^* statistic called *R^2^_LR_* (Sun *et al.* 2010). Finally, the Benjamini and Hochberg (1995) procedure was used to control the false-discovery rate (FDR) at 5 and 10%. Although the latter FDR is less conservative and could hence result in increased false positive signals, it was nevertheless also considered because of the limitations in statistical power arising from the relatively small sample size of the Goodman diversity panel (Lipka *et al.* 2013; Owens *et al.* 2014).

#### Statistical model specific to the NCRPIS panel:

The statistical approach used to evaluate the K_chr model in the NCRPIS panel was similar to that used for the Goodman diversity panel with a few exceptions. To be consistent with the analyses conducted in Romay *et al.* (2013) and Peiffer *et al.* (2014), the VanRaden (2008) method was used to calculate all kinship matrices using a randomly selected 10% of the appropriate subsets of GBS SNPs, and the compressed MLM (Zhang *et al.* 2010) was run in the GAPIT R package (Lipka *et al.* 2012). When the two traits from Romay *et al.* (2013) (*i.e.*, sweet *vs.* starchy and days to silking) were assessed, the first five PCs (from a principal component analysis of the same markers used to calculate the kinship matrices) were included as fixed effect covariates in the Q matrix. To reflect the GWAS models used in Peiffer *et al.* (2014), we substituted the five PCs in the Q matrix with the six eigenvectors exhibiting the largest eigenvalues from the corresponding kinship matrix to evaluate days to anthesis, plant height, and ear height. Finally, to account for the adequate sample size of NCRPIS panel to identify genomic loci associated with complex traits, the Benjamini and Hochberg (1995) procedure was used to control the FDR at only 5%.

### Assessment of performance of the K_chr model relative to the traditional unified MLM

To enable a direct comparison of the K_chr approach to the traditional unified MLM, all traits were also evaluated using the traditional unified MLM as described in Larsson *et al.* (2013), Lipka *et al.* (2013), Romay *et al.* (2013), Peiffer *et al.* (2014), and Owens *et al.* (2014). The results from the traditional unified MLM were compared to the K_chr model in two specific scenarios. First, for each trait with at least one statistically significant marker-trait association found using the K_chr model, the genomic region within ± 250 kb of each significant marker identified from the K_chr model was scanned for significantly associated markers identified (for the same trait) using the traditional unified MLM. In this evaluation, statistical significance was determined at 10% FDR for the Goodman diversity panel (to account for deficiencies in statistical power) and at 5% FDR for the NCRPIS panel. A similar criterion was used to determine if there were any significantly associated markers identified by the traditional unified MLM that were not in the vicinity of markers identified by the K_chr model. Additionally, the distribution of two sets of *P*-values (for a given trait) from markers within specific genomic regions, identified in Lipka *et al.* (2013), Romay *et al.* (2013), and Owens *et al.* (2014) as having peak associations, were compared; one set was from the K_chr model fitted to each marker while the other set was from the traditional unified MLM fitted to each marker. The Wilcoxon signed rank test (Wilcoxon 1945) was then used to compare the resulting two distributions of *P*-values.

### Data availability

To facilitate the implementation of the K_chr model into association studies, we provide sample R code on Github (https://github.com/angelahchen/K_Chr_manuscript).

## Results

### K_chr model tends to identify more significant marker-trait associations than the traditional unified MLM

We explored the ability of the K_chr model proposed by Rincent *et al.* (2014) to detect genomic signals associated with a variety of agronomically important traits measured in two maize diversity panels. Because these traits differ in genetic architecture, we were able to systematically assess the performance of this model for Mendelian (sweet *vs.* starchy), polygenic (carotenoid and tocochromanol), and complex (flowering time and plant height) traits. Furthermore, the contrasting sample sizes of the two diversity panels enabled us to evaluate the performance of the K_chr model for both small and large data sets. For each trait, we compared the number of statistically significant associations identified using the K_chr model to those detected using the traditional unified MLM. At a genome-wide 5% FDR, the K_chr model identified more statistically significant marker-trait associations for the carotenoid and tocochromanol traits (Table 3), as well as for all traits analyzed in the NCRPIS panel except for days to silking (Table 4). To account for the deficiency in the statistical power to detect genomic signals arising from the small sample size of the Goodman diversity panel (Lipka *et al.* 2013; Owens *et al.* 2014), we also compared the number of statistically significant associations detected from the two approaches at a genome-wide 10% FDR. At this FDR, more statistically significant associations for both carotenoids and tocochromanols were also detected using the K_chr model. Collectively, these results suggest that, for Mendelian, polygenic, and complex traits, the K_chr model is capable of detecting a larger number of associated loci compared to the traditional unified MLM.

**Table 3:** Number of significant associations detected at both 5% and 10% false discovery rates between the K_Chr model and traditional unified mixed linear model in the Goodman diversity panel

Trait Class	Genetic Architecture	No. Significant Associations (5% FDR)		No. Significant Associations (10% FDR)		No. Significant Associations Identified Using K_chr Model in Novel Regions***^a^***	No. Significant Associations Identified Using Traditional MLM in Novel Regions***^b^***
		K_Chr	Trad. MLM	K_Chr	Trad. MLM		
Carotenoid	Polygenic	48	30	82	40	28	0
Tocochromanol	Polygenic	110	77	207	146	47	6
Flowering time	Complex	0	0	0	0	0	0

FDR, false discovery rate; MLM, mixed linear model; Trad., traditional.

a A marker that is significantly associated with a trait at 10% FDR when using the K_chr model was declared to be in a novel genomic region if there is no marker within ± 250 kb that is significantly associated with the same trait at 10% FDR when using the traditional unified MLM.

b A marker that is significantly associated with a trait at 10% FDR when using the traditional unified MLM was declared to be in a novel genomic region if there is no marker within ± 250 kb that is significantly associated with the same trait at 10% FDR when using the K_chr model.

**Table 4:** Number of significant associations detected at a 5% false discovery rate between the K_Chr model and traditional unified mixed linear model in the North Central Regional Plant Introduction Station diversity panel

Trait	Genetic Architecture	No. Significant Associations (5% FDR)		No. Significant Associations Identified Using K_chr Model in Novel Regions***^a^***	No. Significant Associations Identified Using Traditional MLM in Novel Regions***^b^***
		K_Chr	Trad. MLM		
Sweet *vs.* starchy	Mendelian	22,600	21,985	18	0
Days to silking	Complex	30,590	32,874	97	0
Days to anthesis	Complex	17,254	11,564	263	0
Plant height	Complex	488	227	33	0
Ear height	Complex	2596	1016	311	0

FDR, false discovery rate; MLM, mixed linear model; Trad., traditional.

a A marker that is significantly associated with a trait at 5% FDR when using the K_chr model was declared to be in a novel genomic region if there is no marker within ± 250 kb that is significantly associated with the same trait at 5% FDR when using the traditional unified MLM.

b A marker that is significantly associated with a trait at 5% FDR when using the traditional unified MLM was declared to be in a novel genomic region if there is no marker within ± 250 kb that is significantly associated with the same trait at 5% FDR when using the K_chr model.

### K_chr model identifies marker-trait associations in novel genomic regions

**Figure 1:**
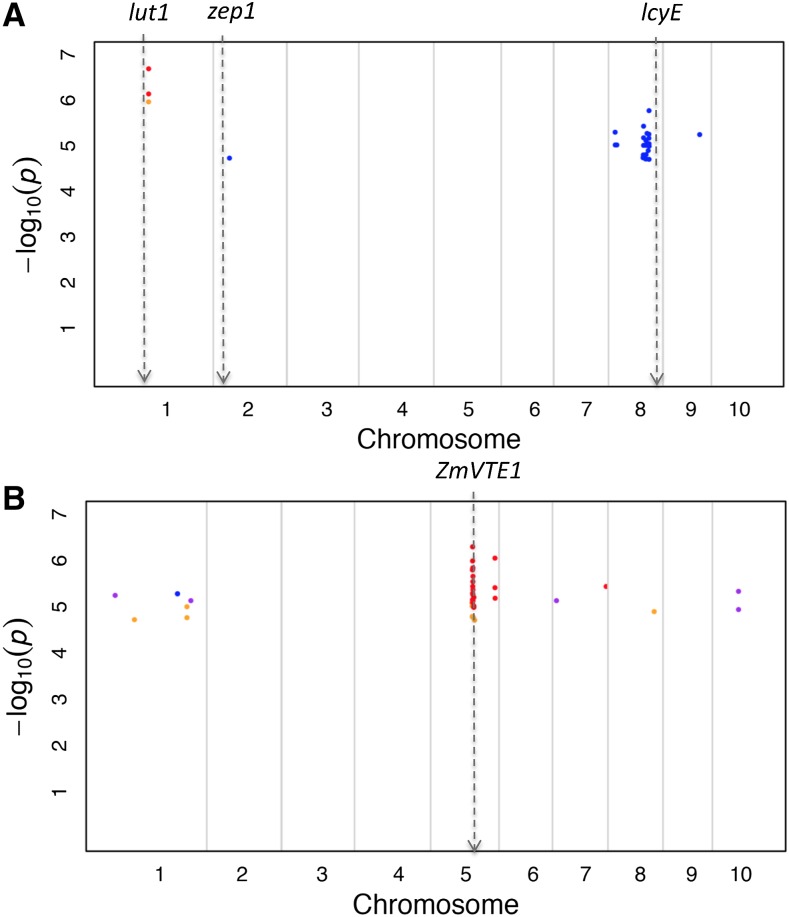
Manhattan plots depicting all SNPs significantly associated with carotenoid (A) and tocochromanol (B) traits at 10% FDR using the K_chr model located in novel genomic regions. Such a SNP is in a novel genomic region if there are no SNPs within ± 250 kb significantly associated with that same trait at 10% FDR when using the traditional unified mixed linear model. (A) The X-axis depicts the B73 RefGen_v2 position along the maize genome and the Y-axis shows the −log(10) *P*-values for each significant SNP at 10% FDR located in a novel genomic region. The blue dots represent novel genomic signals for β-xanthophylls/α-xanthophylls, the light orange dot represents such a signal for α-carotene/zeinoxanthin, and the dark orange dots represent such genomic signals for zeinoxanthin/lutein. The minor allele frequencies of the SNPs depicted in the figure range from 0.09–0.45. (B) The X- and Y-axes are as described in (A). The blue dot represents novel genomic signals for γ-tocopherol/(γ-tocopherol + α-tocopherol), the light orange dots represent such signals for δ-tocotrienol/(γ-tocotrienol + α-tocotrienol), the dark orange dots represent such signals for δ-tocotrienol/γ-tocotrienol, and the purple dots represent such signals for α-tocopherol/γ-tocopherol. The minor allele frequencies of the SNPs depicted in the figure range from 0.08–0.48. The approximate B73 RefGen_v2 positions of relevant biosynthetic pathway genes are depicted by dotted gray arrows. FDR, false discovery rate; SNP, single nucleotide polymorphism.

One critical aspect of this analysis was to determine if the K_chr model detected any genomic signals that were not physically located in the vicinity of those identified using the traditional unified MLM. Thus, for each trait with at least one statistically significant signal detected by the K_chr model, we searched for significant associations identified using the traditional unified MLM (when fitted to the same trait) located within the neighboring ± 250 kb region of each signal detected using the K_chr model. When this approach was used to assess the GWAS results from the Goodman diversity panel, statistical significance was determined at 10% FDR. Accordingly, the K_chr model identified 75 signals located in novel genomic regions that were associated with three carotenoid traits (β-xanthophylls/α-xanthophylls, α-carotene/zeinoxanthin, and zeinoxanthin/lutein) and four tocochromanol traits [γ-tocopherol/(γ-tocopherol + α-tocopherol), δ-tocotrienol/(γ-tocotrienol + α-tocotrienol), δ-tocotrienol/γ-tocotrienol, and α-tocopherol/γ-tocopherol] (Figure 1, Table 3, and Table 5). Under a similar criterion, the traditional unified MLM identified six signals associated with three tocochromanol traits (α-tocopherol, δ-tocopherol/α-tocopherol, and α-tocopherol/γ-tocopherol) that were located in novel genomic regions (Figure S1, Table 3, and Table S2).

**Table 5:** For each indicated trait analyzed in the Goodman diversity panel, the number of significant markers identified by the K_chr model at 10% false discovery rate that are located in novel genomic regions are presented

Trait Name	No. Significant Associations in Novel Regions*^a^*	B73 RefGen_v2 Position of Nearest Novel Significant Association to Candidate Gene*^b^*	Candidate Gene Name and B73 RefGen_v2 Position*^c^*
β-Xanthophylls/α-xanthophylls	25	Chr 2: 51,751,723	*zep1* - Chr2: 44,440,299-44,449,237
		Chr 8: 131,533,827	*lcyE* - Chr8: 138,882,594-138,889,812
α-Carotene/zeinoxanthin	1	Chr 1: 92,347,976	*lut1* - Chr1: 86,838,334-86,848,726
Zeinoxanthin/lutein	2	Chr 1: 92,347,976	*lut1* - Chr1: 86,838,334-86,848,726
γ-Tocopherol/(γ-tocopherol + α-tocopherol)	2	NA	NA
δ-Tocotrienol/(γ-tocotrienol + α-tocotrienol)	10	Chr 5: 132,656,905	*ZmVTE1* - Chr 5: 133,501,928-133,518,495
δ-Tocotrienol/γ-tocotrienol	30	Chr 5: 133,501,858	*ZmVTE1* - Chr 5: 133,501,928 - 133,518,495
α-Tocopherol/γ-tocopherol	5	NA	NA

For all such markers that are on the same chromosome as an *a priori* candidate gene, information about the corresponding candidate gene is provided. Chr., chromosome; NA, not applicable.

a A marker that is significantly associated with a trait at 10% false discovery rate (FDR) when using the K_chr model was declared to be in a novel genomic region if there is no marker within ± 250 kb that is significantly associated with the same trait at 10% FDR when using the traditional unified mixed linear model (MLM).

b If at least one of the markers significantly associated with a trait at 10% FDR using the K_chr model is located in a novel genomic region on the same chromosome as a relevant candidate gene, then the B73 RefGen_v2 position of the closest such marker to the candidate gene is reported.

c When applicable, the name of the nearest candidate gene (as depicted in Owens *et al.* 2014 and Lipka *et al.* 2013) as well as their B73 RefGen_v2 ORF (open reading frame) start and stop bp are reported.

**Figure 2:**
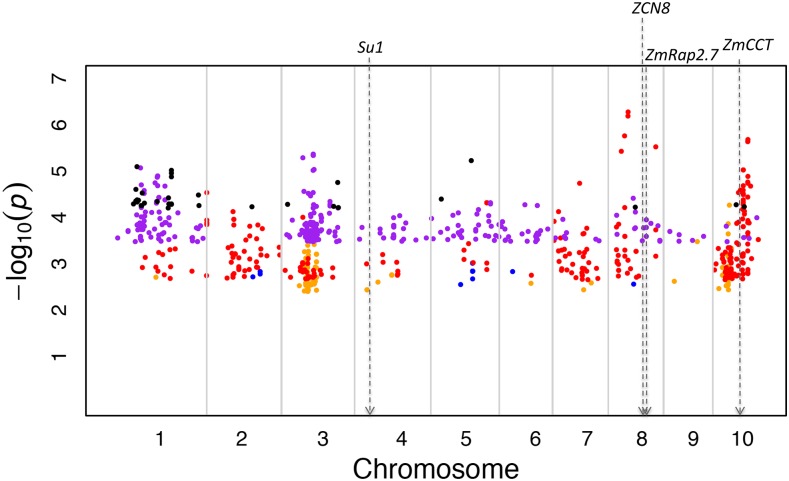
Manhattan plot depicting all SNPs significantly associated with the traits evaluated in the North Central Regional Plant Introduction Station panel at 5% FDR using the K_chr model located in novel genomic regions. Such a SNP is in a novel genomic region if there are no SNPs within ± 250 kb significantly associated with that same trait at 5% FDR when using the traditional unified mixed linear model. The X-axis depicts the B73 RefGen_v2 position along the maize genome and the Y-axis shows the −log(10) *P*-values for each significant SNP at 5% FDR located in a novel genomic region. The blue dots represent novel genomic signals for sweet *vs.* starchy corn, the light orange dots represent such signals for days to silking, the red dots represent such signals for days to anthesis, the black dots represent such signals for plant height, and the purple dots represent such signals for ear height. The minor allele frequencies of the SNPs depicted in the figure range from 0.05–0.50. The approximate B73 RefGen_v2 positions of relevant candidate genes and regulatory elements are depicted by dotted gray arrows. FDR, false discovery rate; SNP, single nucleotide polymorphism.

When this analysis was conducted in the NCRPIS panel, statistical significance was determined at 5% FDR. Across the five traits assessed in this panel, the K_chr model detected a total of 722 signals located in novel genomic regions (Figure 2, Table 4, and Table 6). These signals were distributed throughout the entire genome, the strongest of which was located on chromosome 8 (associated with days to anthesis at *P*-value 5.26 × 10^−7^; Figure 2). In contrast to the K_chr model, no statistically significant signals located in novel genomic regions were identified in the NCRPIS panel using the traditional unified MLM.

**Table 6:** For each indicated trait analyzed in the North Central Regional Plant Introduction Station panel, the number of significant markers identified by the K_chr model at 5% false discovery rate that are located in novel genomic regions are presented

Trait Name	No. Significant Associations in Novel Regions*^a^*	B73 RefGen_v2 Position of Nearest Novel Significant Association to Candidate Gene*^b^*	Candidate Gene/Regulatory Element Name and B73 RefGen_v2 Position*^c^*
Sweet *vs.* starchy	18	NA	NA
Days to silking	97	Chr 10: 58,673,233	*ZmCCT* - Chr10: 94,248,710-94,251,264
Days to anthesis	263	Chr 8: 96,929,838	*ZCN8* - Chr8: 123,501,085-123,502,873
		Chr 8: 150,876,807	*ZmRap2.7* - Chr8: 132,044,001
		Chr 10: 94,588,819	*ZmCCT* - Chr10: 94,248,710-94,251,264
Plant height	33	NA	NA
Ear height	311	NA	NA

For all such markers that are on the same chromosome as an *a priori* candidate gene or regulatory element, corresponding genomic information is provided. NA, not applicable; Chr., chromosome.

a A marker that is significantly associated with a trait at 5% false discovery rate (FDR) when using the K_chr model was declared to be in a novel genomic region if there is no marker within ± 250 kb that is significantly associated with the same trait at 5% FDR when using the traditional unified MLM.

b If at least one of the markers significantly associated with a trait at 5% FDR using the K_chr model is located in a novel genomic region on the same chromosome as a relevant candidate gene or regulatory element, then the B73 RefGen_v2 position of the closest such marker is reported.

c When applicable, the name of the nearest candidate gene or regulatory element as well as their B73 RefGen_v2 ORF (open reading frame) start and stop bp are reported.

Across all of the traits in which the K_chr model identified signals in novel genomic regions, δ-tocotrienol/γ-tocotrienol (which was analyzed in the Goodman diversity panel) had associations in a novel chromosome 5 genomic region that completely encompassed the tocochromanol biosynthetic pathway gene *ZmVTE1*. Within this region, the peak SNP locus (S5_133501858; 133,501,858 bp; *P*-value 4.98 × 10^−7^) was located only 70 bp from the *ZmVTE1* transcriptional start site. This result is particularly interesting because no statistically significant marker-trait associations were identified for this trait when the GWAS was conducted using the traditional unified MLM (Lipka *et al.* 2013). Although the biological basis of these novel signals needs to be rigorously evaluated in future molecular biology and biochemical studies, these findings nevertheless indicate that the K_chr model is capable of detecting genomic sources of phenotypic variation on a finer scale relative to the traditional unified MLM.

### K_chr model provides more insight into genomic signals in high LD regions

We next evaluated the ability of the K_chr model to further elucidate the sources of genomic variation underlying specific regions harboring peak GWAS associations for five carotenoid traits (zeinoxanthin, β-xanthophylls/α-xanthophylls, α-carotene/zeinoxanthin, zeinoxanthin/lutein, and zeaxanthin), seven tocochromanol traits [δ -tocotrienol/γ-tocotrienol, α-tocopherol, δ-tocotrienol, δ-tocotrienol/(γ-tocotrienol + α-tocotrienol), δ-tocopherol/α-tocopherol, γ-tocopherol/(γ-tocopherol + α-tocopherol), and α-tocopherol/γ-tocopherol], and all five of the traits analyzed in the NCRPIS panel. All of these genomic regions have been identified in previous association studies (Lipka *et al.* 2013; Owens *et al.* 2014; Romay *et al.* 2013). Because the amount of local LD varied among these genomic regions (as reported in Lipka *et al.* 2013, Owens *et al.* 2014, and Romay *et al.* 2013), our evaluation enabled a direct comparison of the performance of the K_chr model to the traditional unified MLM under various levels of local LD decay. To ensure a rigorous assessment of the impact of local LD on the performance of the K_chr model, three genomic regions in high LD (*i.e.*, the chromosome 1 region containing *lut1*, the chromosome 4 region containing *Su1*, and the chromosome 5 region containing *ZmVTE1*) and three genomic regions in lower LD (the chromosome 2 region containing *zep1*, the chromosome 5 region containing *ZmVTE4*, and the chromosome 8 region containing *ZCN8* and *ZmRap2.7*) were assessed.

**Figure 3:**
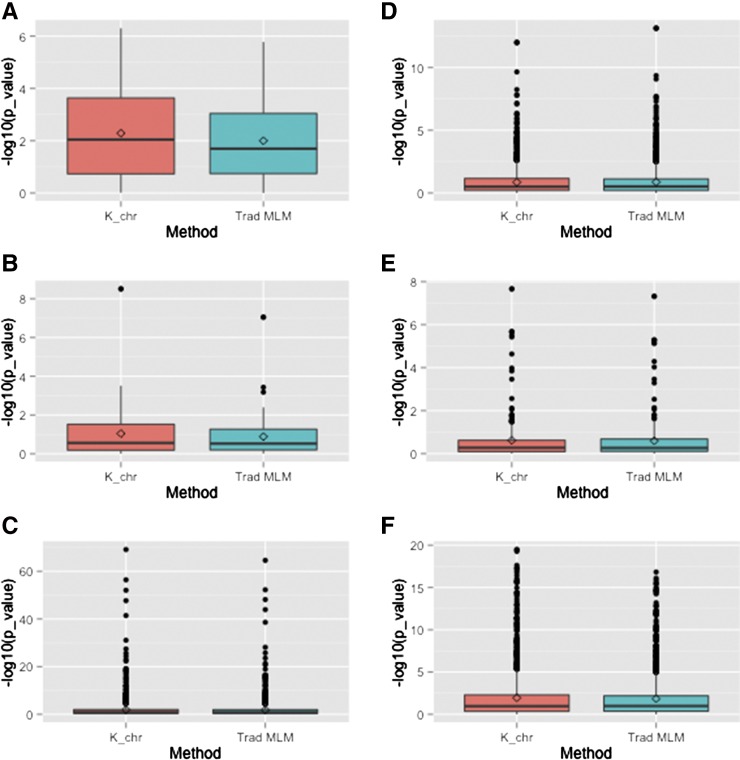
Distribution of *P*-values obtained from the K_chr model and traditional unified mixed linear model (MLM) at six specific genomic regions, each of which contain at least one candidate gene. Each graph compares the distribution of *P*-values from the K_chr model (red box plot, left) to those from the traditional unified MLM (blue box plot, right). The −log(10) *P*-values are presented on the *Y*-axis. (A) Distribution of *P*-values from the K_chr model and MLM when markers within the chromosome 5 region surrounding *ZmVTE1* were tested for association with δ-tocotrienol/γ-tocotrienol. (B) Distribution of *P*-values from the K_chr model and MLM when markers in the chromosome 1 region surrounding *lut1* were tested for association with zeinoxanthin. (C) Distribution of *P*-values from the K_chr model and MLM when markers in the chromosome 4 region surrounding *Su1* were tested for associations with sweet *vs.* starchy corn. (D) Distribution of *P*-values from the K_chr model and MLM when markers in the chromosome 5 region surrounding *ZmVTE4* were tested for associations with α-tocopherol. (E) Distribution of *P*-values from the K_chr model and MLM when markers in the chromosome 2 region surrounding *zep1* were tested for associations with β-xanthophylls/α-xanthophylls. (F) Distribution of *P*-values from the K_chr model and MLM when markers in the chromosome 8 region surrounding *ZCN8* and *ZmRap2.7* were tested for associations with days to silking. For the regions with high local linkage disequilibrium (LD; *i.e.*, those presented in A, B, and C), the distribution of *P*-values from the K_chr model are noticeably lower than the distribution presented by the traditional unified MLM. The same trend is observed for the two regions analyzed using data from the powerful North Central Regional Plant Introduction Station panel (presented in C and F). Finally, the distribution of *P*-values from the two different models are more similar in regions of lower LD (presented in D and E) analyzed using data from the smaller Goodman diversity panel.

Within each of these six genomic regions, we compared the distribution of *P*-values obtained from the K_chr model to those obtained using the traditional unified MLM for all markers located inside the region (Figure 3, Figure S2, Figure S3, Figure S4, Figure S5, Figure S6, Table 7, and Table 8). For the three genomic regions in high LD, the K_chr model appeared to yield a distribution of more significant *P*-values relative to the traditional unified MLM (Wilcoxon signed rank test *P*-values ranging from < 2.20 × 10^−16^ to 5.29 × 10^−2^). A similar result was obtained for the chromosome 8 region harboring significant associations for the flowering time- and plant height-related traits evaluated in the powerful NCRPIS panel. In contrast, the distribution of the *P*-values from the two approaches did not differ as much for the markers that are located within the genomic regions containing *zep1* and *ZmVTE4* (Wilcoxon signed rank test *P*-value ranging from 4.29 × 10^−2^ to 8.69 × 10^−1^). Thus, our findings are consistent with those presented Rincent *et al.* (2014), namely that the K_chr model shows a clear advantage in power over the traditional unified MLM in regions of high LD.

**Table 7:** For each genomic region surrounding the indicated *a priori* candidate gene that was assessed using results from the Goodman diversity panel, the Wilcoxon signed rank test was used to compare the distribution of *P*-values obtained from the K_chr model to those from the traditional unified mixed linear model

*ZmVTE1*		*lut1*		*ZmVTE4*		*zep1*	
Trait Analyzed	*P*-Value	Trait Analyzed	*P*-Value	Trait Analyzed	*P*-Value	Trait Analyzed	*P*-Value
δ-Tocotrienol	< 2.20 × 10^−16^	Zeinoxanthin	5.29 × 10^−2^	α-Tocopherol	8.69 × 10^−1^	Zeaxanthin	2.17 × 10^−1^
δ-Tocotrienol/(γ-tocotrienol + α-tocotrienol)	< 2.20 × 10^−16^	α-Carotene/zeinoxanthin	1.25 × 10^−3^	δ-Tocopherol/α-tocopherol	4.29 × 10^−2^	β-Xanthophylls/α-xanthophylls	1.16 × 10^−1^
δ-Tocotrienol/γ-tocotrienol	< 2.20 × 10^−16^	Zeinoxanthin/lutein	4.45 × 10^−3^	γ-Tocopherol/(γ-tocopherol + α-tocopherol)	7.30 × 10^−1^		
				α-Tocopherol/γ-tocopherol	2.5 × 10^−1^		

For each indicated trait, *P*-values from the Wilcoxon signed rank test are reported. The genomic regions surrounding *ZmVTE1* and *lut1* are in high linkage disequilibrium (LD), while the genomic regions surrounding *ZmVTE4* and *zep1* are in lower LD.

**Table 8:** For each genomic region surrounding the indicated *a priori* candidate gene or regulatory element that was assessed using results from the North Central Regional Plant Introduction Station panel, the Wilcoxon signed rank test is used to compare the distribution of *P*-values obtained from the K_chr model to those from the traditional unified mixed linear model

*Su1*		*ZCN8* and *ZmRap2.7*	
Trait Analyzed	*P*-Value	Trait Analyzed	*P*-Value
Sweet *vs.* starchy	< 2.20 × 10^−16^	Days to anthesis	< 2.20 × 10^−16^
		Days to silking	< 2.20 × 10^−16^
		Plant height	< 2.20 × 10^−16^
		Ear height	< 2.20 × 10^−16^

For each indicated trait, *P*-values from the Wilcoxon signed rank test are reported. The genomic region surrounding *Su1* is in high linkage disequilibrium (LD), while the genomic region surrounding *ZCN8* and *ZmRap2.7* is in lower LD.

## Discussion

We compared the performance of the K_chr model to the traditional unified MLM by running a GWAS on a series of traits contrasting in genetic architecture in two maize diversity panels. This study was conducted because the K_chr model appears to be an effective approach for increasing the ability to detect marker-trait associations, while still controlling for population structure and relatedness in the mixed model framework originally proposed in Yu *et al.* (2006). We clearly demonstrated that the K_chr model is capable of finding more statistically significant marker-trait associations than the traditional unified MLM. In high LD genomic regions where significant signals were found using the traditional unified MLM (as reported in Lipka *et al.* 2013, Romay *et al.* 2013, and Owens *et al.* 2014), the K_chr model generally yielded lower *P*-values when fitted to the surrounding markers. This result is exemplified by the novel detection of significant associations between markers in a high LD genomic region containing the tocochromanol biosynthetic pathway gene *ZmVTE1* and δ-tocotrienol/γ-tocotrienol. Because the traits we evaluated encompass a wide variety of fundamental characteristics of maize, our results suggest that the use of the K_chr model to identify marker-trait associations could have important nutritional and agronomical implications.

The discrepancy in the ability of the two panels to detect significant marker-trait associations is best illustrated by comparing the results for the flowering time-related traits (Table 3 and Table 4). Because of its complex genetic architecture in maize (Buckler *et al.* 2009), detection of the weak genomic signals underlying flowering time is inherently difficult. This issue is potentially exacerbated in the Goodman diversity panel because of its relatively small sample size. Furthermore, the density of the marker sets we analyzed was most likely insufficient to cover the LD patterns of the entire genome, especially given the rapid LD decay in maize (Remington *et al.* 2001). All of these factors are likely to have contributed to our inability to discover statistically significant associations for the flowering time-related traits in the Goodman diversity panel. In contrast, both GWAS models identified tens of thousands of statistically significant marker-trait associations for the two flowering time-related traits evaluated in the NCRPIS panel (*i.e.*, days to silking and days to anthesis; Table 4). The aspect that most likely contributed to this result was the increased statistical power available in the 2815-member NCRPIS panel. Indeed, we were also able to identify significant associations for another two complex traits (plant height and ear height), as well as a Mendelian trait (sweet *vs.* starchy corn) in this panel. Thus, the most important ramification of the GWAS conducted in the NCRPIS panel was that it demonstrated the potential of the K_chr model to provide further elucidation of the genomic sources of complex and Mendelian traits.

Using specific genomic regions identified in previous studies to have peak associations with sweet *vs.* starchy corn, carotenoid traits, and tocochromanol traits (Lipka *et al.* 2013; Owens *et al.* 2014; Romay *et al.* 2013), we were able to show that the K_chr model can find more significant marker-trait associations than the traditional unified MLM in high-LD genomic regions. In agreement with the findings of Rincent *et al.* (2014), our results suggest that the K_chr model should be considered to be a fundamental approach that could provide the resolution needed to go after elusive sources of genomic variation located in pericentromeric and other regions of recombination suppression. Additionally, our analysis of the flowering time- and plant height-related results in the chromosome 8 region containing *ZCN8* and *ZmRap2.7* suggest that the K_chr model can further refine genomic signals in lower LD regions if the sample size is sufficiently large. Thus, we recommend using the K_chr model as a starting point for any association analysis: after a genomic signal in a high-LD region is identified with the K_chr model, a chromosome-wide stepwise multi-locus mixed model (MLMM; Segura *et al.* 2012) should be conducted on the chromosome where the signal was identified using the same kinship matrix that was implemented in the K_chr model. By using this kinship matrix (instead of the genome-wide kinship matrix typically employed in the MLMM), it is likely that the MLMM will have greater power to conduct a more exhaustive search for multiple loci underlying the signal originally detected by the K_chr model.

Interestingly, a sizeable proportion of the signals detected by the K_chr model were not in the vicinity of those found by the traditional unified MLM (Figure 1, Figure 2, Table 3, and Table 4). Although the criterion we used to determine which signals identified using the K_chr model were located in novel genomic regions was based on those from previous maize studies (Salvi *et al.* 2007; Lipka *et al.* 2013; Owens *et al.* 2014), other equally biologically valid criteria could be used to determine which signals are in novel genomic regions. Furthermore, these other criteria could result in different conclusions about the proportion of K_chr signals that reside in novel genomic regions. Coupled with the fact that long-range LD between markers and ungenotyped causal mutations could lead to false conclusions about the location of true genomic signals (Platt *et al.* 2010; Dickson *et al.* 2010), our criterion should only be interpreted as a rough approximation of the amount of novel genomic regions detected by the K_chr model. As such, we recommend interpreting our results on the proportion of significant K_chr associations in novel genomic regions within the context of the total number of additional significant signals identified using the K_chr approach. Nevertheless, we were able to use the K_chr model to detect novel genomic signals on chromosome 5 that were significantly associated with δ-tocotrienol/γ-tocotrienol. Because these signals encompass the tocochromanol biosynthetic pathway gene *ZmVTE1*, the identification of significant marker-trait associations in this region makes sense from a biochemical perspective. If these novel associations detected using the K_chr approach can be biologically validated, then our findings could lead to a more complete understanding of tocotrienol biosynthesis in maize grain.

The more widespread implementation of the K_chr model in association studies could have a profound impact on the ability to detect biologically significant marker-trait associations, especially those that reside in high LD regions. Compared to the traditional unified MLM, the K_chr model is likely to find more intricate sources of variation underlying a genomic signal, which could pave the way toward identifying a greater number of causal mutations and haplotypes responsible for the peak marker-trait associations. Because of this, the use of the K_chr model could lead to the discovery of novel sources of allelic variation that could both enhance our understanding of the genetic architecture of important traits and lead to the elucidation of novel targets for marker-assisted selection. 

## Supplementary Material

Supplemental Material
