# Supplementary material for: The Use of Targeted Marker Subsets to Account for Population Structure and Relatedness in Genome-Wide Association Studies of Maize (Zea mays L.)
Source: G3 (Bethesda). 2016 May 26;6(8):2365–74. doi: 10.1534/g3.116.029090 (PMC4978891; doi:10.1534/g3.116.029090)
Supplement: Supplemental Material [file supp_6_8_2365__index.html]

The Use of Targeted Marker Subsets to Account for Population Structure and Relatedness in Genome-Wide Association Studies of Maize (Zea mays L.) — Supplemental Material 

# The Use of Targeted Marker Subsets to Account for Population Structure and Relatedness in Genome-Wide Association Studies of Maize (*Zea mays* L.)

## Supplemental Material for Chen and Lipka, 2016

**Files in this Data Supplement:**

- Table S1 - (a) Summary Statistics for the three marker sets used in the Goodman diversity panel. (b) Summary Statistics for the genotyping-by-sequencing GBS markers used in the North Central Regional Plant Introduction Station (NCRPIS) panel. (.pdf, 208 KB)
- Table S2 - For each indicated trait analyzed in the Goodman diversity panel, the number of significant markers identified by the traditional unified mixed linear model (MLM) model at 10% false discovery rate that are located in novel genomic regions are presented. (.pdf, 183 KB)
- Figure S1 - Manhattan plots depicting all SNPs significantly associated with three tocochromanol traits at 10% false discovery rate (FDR) using the traditional unified mixed linear model (MLM) located in novel genomic regions. (.pptx, 158 KB)
- Figure S2 - Distribution of *P*-values obtained from the K\_chr and traditional unified mixed linear models at all markers located within a chromosome 5 genomic region surrounding the tocochromanol biosynthetic pathway gene *ZmVTE1*. (.pptx, 1,013 KB)
- Figure S3 - Distribution of P-values obtained from the K\_chr and traditional unified mixed linear models at all markers located within a chromosome 1 genomic region surrounding the carotenoid biosynthetic pathway gene *lut1*. (.pptx, 1,012 KB)
- Figure S4 - Distribution of P-values obtained from the K\_chr and traditional unified mixed linear models at all markers located within a chromosome 5 genomic region surrounding the tocochromanol biosynthetic pathway gene *ZmVTE4*. (.pptx, 1,495 KB)
- Figure S5 - Distribution of P-values obtained from the K\_chr and traditional unified mixed linear models at all markers located within a chromosome 2 genomic region surrounding the carotenoid biosynthetic pathway gene *zep1* when tested for an association with zeaxanthin. (.pptx, 527 KB)
- Figure S6 - Distribution of P-values obtained from the K\_chr and traditional unified mixed linear models at all markers located within a chromosome 8 region surrounding *ZCN8* and *ZmRap2.7*. (.pptx, 100 KB)
