# Supplementary material for: The Use of Targeted Marker Subsets to Account for Population Structure and Relatedness in Genome-Wide Association Studies of Maize (Zea mays L.)
Source: G3 (Bethesda). 2016 May 26;6(8):2365–74. doi: 10.1534/g3.116.029090 (PMC4978891; doi:10.1534/g3.116.029090)
Supplement: Supplemental Material [file supp_g3.116.029090_TableS1.pdf]

Table S1a. Summary Statistics for the three marker sets used in the Goodman diversity panel.

Stat Type	Value 50K[a]	Value Other SNPs[b]	Value GBS Chr1[c]	Value GBS Chr2	Value GBS Chr3	Value GBS Chr4	Value GBS Chr5	Value GBS Chr6	Value GBS Chr7	Value GBS Chr8	Value GBS Chr9	Value GBS Chr10
Number of Taxa	280	281	282	282	282	282	282	282	282	282	282	282
Number of Sites	51741	3093	64978	50093	47577	35942	47364	33426	34453	35049	30162	28824
Sites x Taxa	1.45E+07	869133	1.83E+07	1.41E+07	1.34E+07	1.01E+07	1.34E+07	9.43E+06	9.72E+06	9.88E+06	8.51E+06	8.13E+06
Number Not Missing	1.38E+07	837722	1.53E+07	1.18E+07	1.12E+07	8.61E+06	1.13E+07	7.83E+06	8.17E+06	8.18E+06	7.14E+06	6.84E+06
Proportion Not Missing	0.95323	0.96386	0.83518	0.83853	0.83565	0.84967	0.84372	0.83028	0.84101	0.82756	0.83943	0.84121
Number Missing	677527	31411	3.02E+06	2.28E+06	2.21E+06	1.52E+06	2.09E+06	1.60E+06	1.54E+06	1.70E+06	1.37E+06	1.29E+06
Proportion Missing	0.04677	0.03614	0.16482	0.16147	0.16435	0.15033	0.15628	0.16972	0.15899	0.17244	0.16057	0.15879
Number Gametes	2.90E+07	1.74E+06	3.66E+07	2.83E+07	2.68E+07	2.03E+07	2.67E+07	1.89E+07	1.94E+07	1.98E+07	1.70E+07	1.63E+07
Gametes Not Missing	2.76E+07	1.68E+06	3.06E+07	2.37E+07	2.24E+07	1.72E+07	2.25E+07	1.57E+07	1.63E+07	1.64E+07	1.43E+07	1.37E+07
Proportion Gametes Not Missing	0.95323	0.96386	0.83518	0.83853	0.83565	0.84967	0.84372	0.83028	0.84101	0.82756	0.83943	0.84121
Gametes Missing	1.36E+06	62822	6.04E+06	4.56E+06	4.41E+06	3.05E+06	4.17E+06	3.20E+06	3.09E+06	3.41E+06	2.73E+06	2.58E+06
Proportion Gametes Missing	0.04677	0.03614	0.16482	0.16147	0.16435	0.15033	0.15628	0.16972	0.15899	0.17244	0.16057	0.15879
Number Heterozygous	49034	9622	18012	14985	13095	9971	13180	9281	9534	9707	9239	9079
Proportion Heterozygous	0.00338	0.01107	9.83E-04	0.00106	9.76E-04	9.84E-04	9.87E-04	9.85E-04	9.81E-04	9.82E-04	0.00109	0.00112

[a]Refers to the markers in the MaizeSNP50 BeadChip

[b]Refers to the markers obtained from several SNP genotyping assays, as described in the Materials and Methods

[b]GBS, genotyping-by-sequencing

Table S1b. Summary Statistics for the genotyping-by-sequencing GBS markers used in the North Central Regional Plant Introduction Station (NCRPIS) panel.

Stat Type	Value GBS Chr 1	Value GBS Chr 2	Value GBS Chr 3	Value GBS Chr 4	Value GBS Chr 5	Value GBS Chr 6	Value GBS Chr 7	Value GBS Chr 8	Value GBS Chr 9	Value GBS Chr 10
Number of Taxa	4476	4476	4476	4476	4476	4476	4476	4476	4476	4476
Number of Sites	108292	82006	77207	66512	77067	55489	57504	58568	50473	48139
Sites x Taxa	4.85E+08	3.67E+08	3.46E+08	2.98E+08	3.45E+08	2.48E+08	2.57E+08	2.62E+08	2.26E+08	2.15E+08
Number Not Missing	4.41E+08	3.31E+08	3.10E+08	2.73E+08	3.13E+08	2.25E+08	2.35E+08	2.36E+08	2.06E+08	1.96E+08
Proportion Not Missing	0.90897	0.90217	0.89669	0.9179	0.90713	0.90484	0.91123	0.89926	0.91282	0.90874
Number Missing	4.41E+07	3.59E+07	3.57E+07	2.44E+07	3.20E+07	2.36E+07	2.28E+07	2.64E+07	1.97E+07	1.97E+07
Proportion Missing	0.09103	0.09783	0.10331	0.0821	0.09287	0.09516	0.08877	0.10074	0.08718	0.09126
Number Gametes	9.69E+08	7.34E+08	6.91E+08	5.95E+08	6.90E+08	4.97E+08	5.15E+08	5.24E+08	4.52E+08	4.31E+08
Gametes Not Missing	8.81E+08	6.62E+08	6.20E+08	5.47E+08	6.26E+08	4.49E+08	4.69E+08	4.71E+08	4.12E+08	3.92E+08
Proportion Gametes Not Missing	0.90897	0.90217	0.89669	0.9179	0.90713	0.90484	0.91123	0.89926	0.91282	0.90874
Gametes Missing	8.83E+07	7.18E+07	7.14E+07	4.89E+07	6.41E+07	4.73E+07	4.57E+07	5.28E+07	3.94E+07	3.93E+07
Proportion Gametes Missing	0.09103	0.09783	0.10331	0.0821	0.09287	0.09516	0.08877	0.10074	0.08718	0.09126
Number Heterozygous	436909	350130	316707	242106	313306	229276	223722	236625	207392	207727
Proportion Heterozygous	9.01E-04	9.54E-04	9.16E-04	8.13E-04	9.08E-04	9.23E-04	8.69E-04	9.03E-04	9.18E-04	9.64E-04
