# Supplementary material for: The Use of Targeted Marker Subsets to Account for Population Structure and Relatedness in Genome-Wide Association Studies of Maize (Zea mays L.)
Source: G3 (Bethesda). 2016 May 26;6(8):2365–74. doi: 10.1534/g3.116.029090 (PMC4978891; doi:10.1534/g3.116.029090)
Supplement: Supplemental Material [file supp_g3.116.029090_TableS2.pdf]

Table S2. For each indicated trait analyzed in the Goodman diversity panel, the number of significant markers identified by the traditional unified mixed linear model (MLM) model at 10% false discovery rate that are located in novel genomic regions are presented. For all such markers that are on the same chromosome an *a priori* candidate gene, information about the corresponding candidate gene is provided.

Trait Name	No. Significant Associations in Novel Regions[a]	B73 RefGen v2 Position of Nearest Novel Significant Association to Candidate Gene[b]	Candidate Gene Name and B73 RefGen v2 Position[c]
α-Tocopherol	2	Chr 5: 199,647,093	*ZmVTE4* - Chr 5: 200,367,029-200,370,851 bp
δ-Tocopherol/α-tocopherol	3	NA	NA
α-Tocopherol/γ-tocopherol	1	NA	NA

[a]A marker that is significantly associated with a trait at 10% false discovery rate (FDR) when using the traditional unified MLM was declared to be in a novel genomic region if there is no marker within +/- 250 kb that is significantly associated with the same trait at 10% FDR when using the K_chr model.

[b]If at least one of the markers significantly associated with a trait at 10% FDR using the traditional unified MLM is located in a novel genomic region on the same chromosome as a relevant candidate gene, then the B73 RefGen v2 position of the closest such marker to the candidate gene is reported.

[c]When applicable, the name of the nearest candidate gene (as depicted in Owens et al. 2014 and Lipka et al. 2013) as well as their B73 RefGen v2 ORF start and stop bp are reported.
