# Supplementary figures and images for: The Use of Targeted Marker Subsets to Account for Population Structure and Relatedness in Genome-Wide Association Studies of Maize (Zea mays L.)
Source: G3 (Bethesda). 2016 May 26;6(8):2365–74. doi: 10.1534/g3.116.029090 (PMC4978891; doi:10.1534/g3.116.029090)

## Slide 1
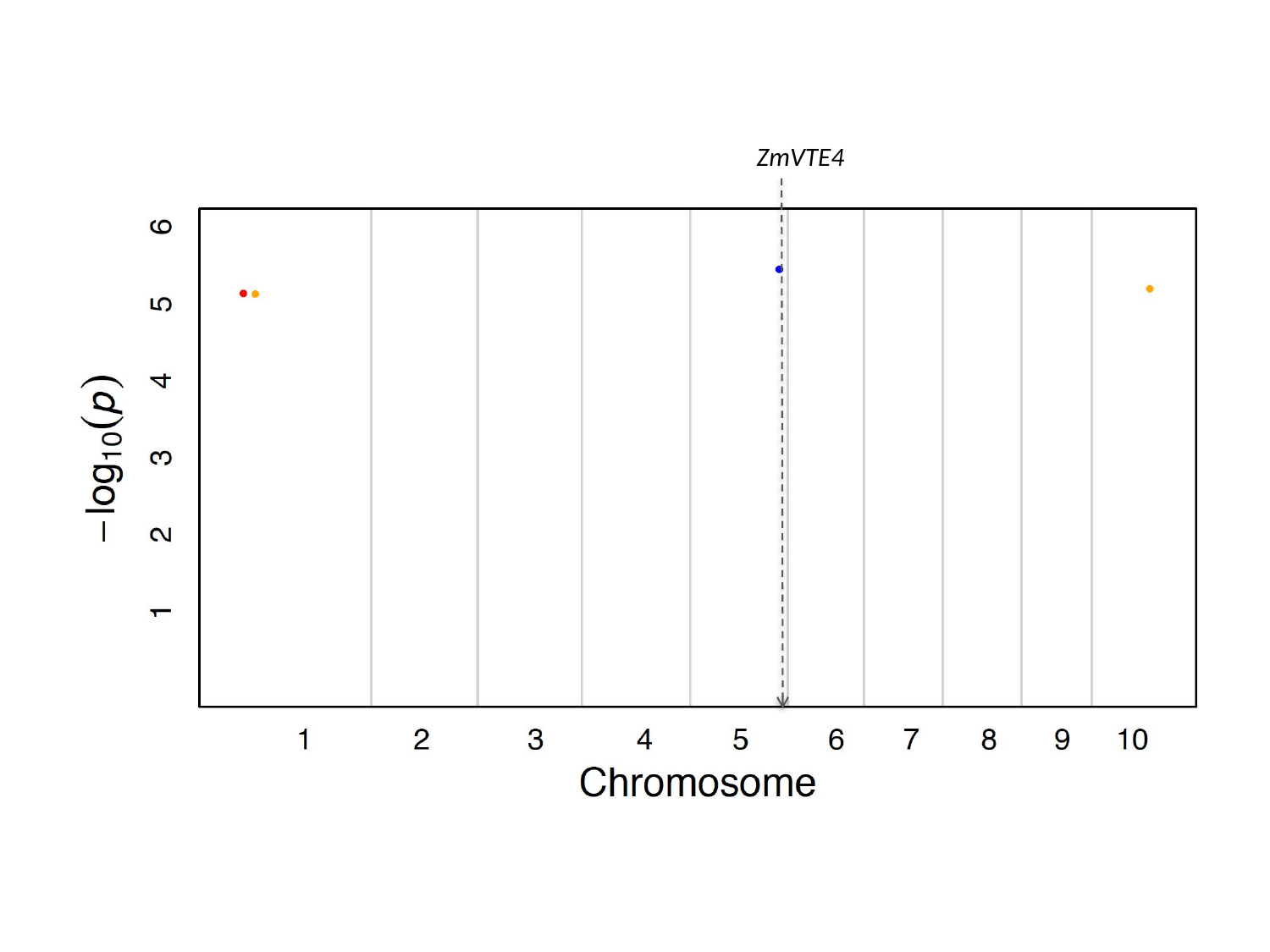

ZmVTE4

Supplement: Supplemental Material [file supp_g3.116.029090_FigureS1.pptx]

## Slide 1
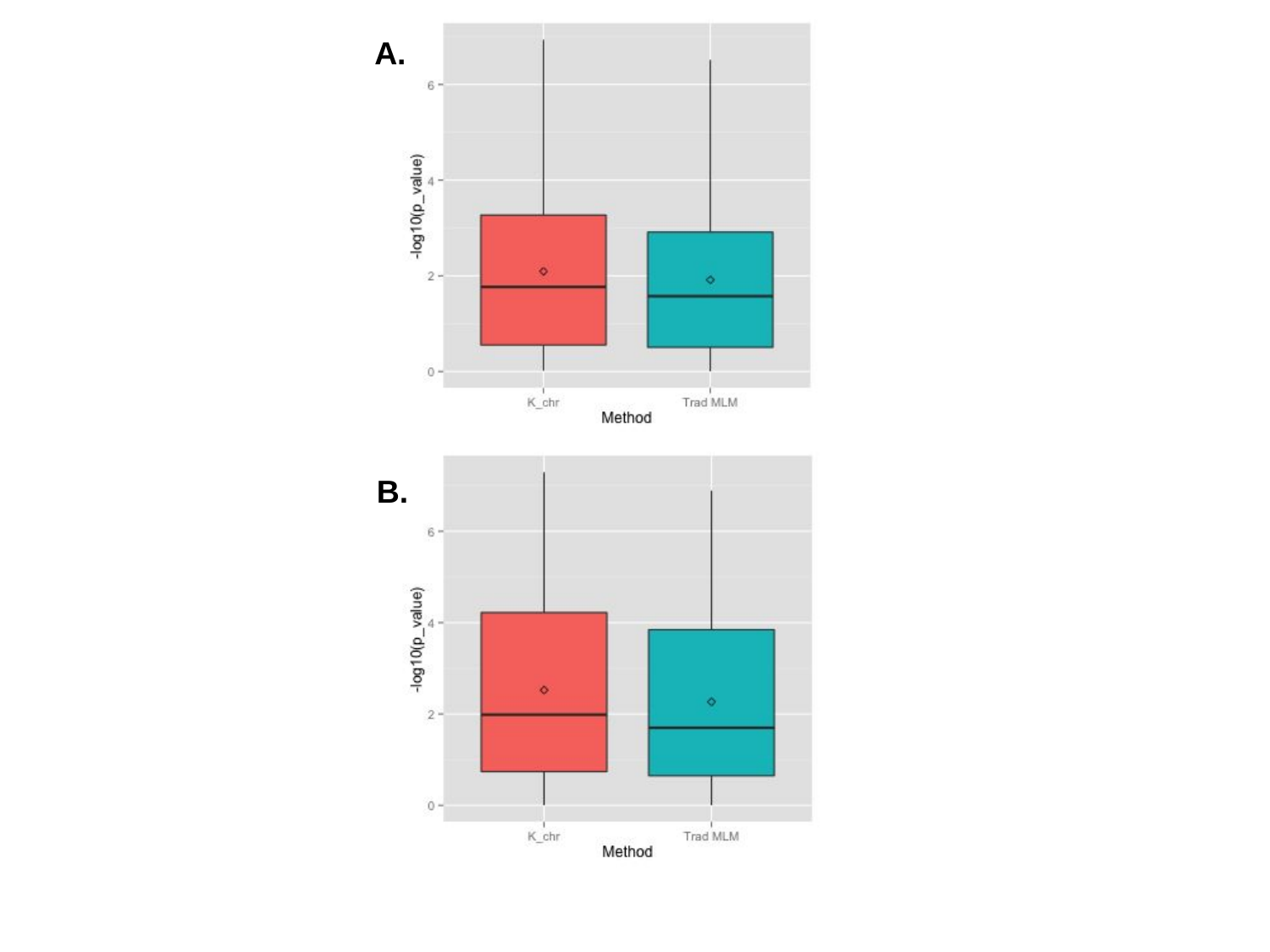

A.
B.

Supplement: Supplemental Material [file supp_g3.116.029090_FigureS2.pptx]

## Slide 1
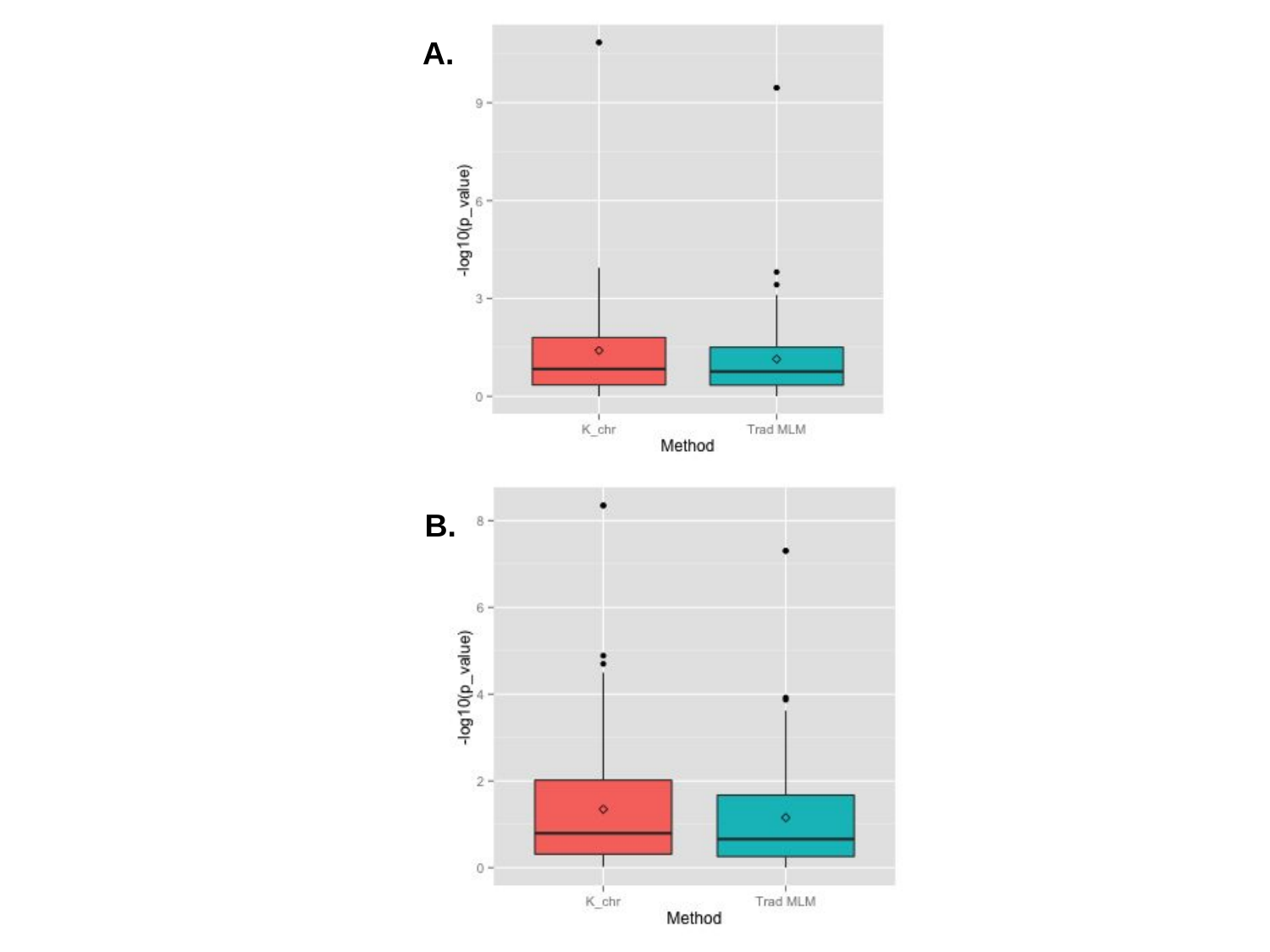

A.
B.

Supplement: Supplemental Material [file supp_g3.116.029090_FigureS3.pptx]

## Slide 1
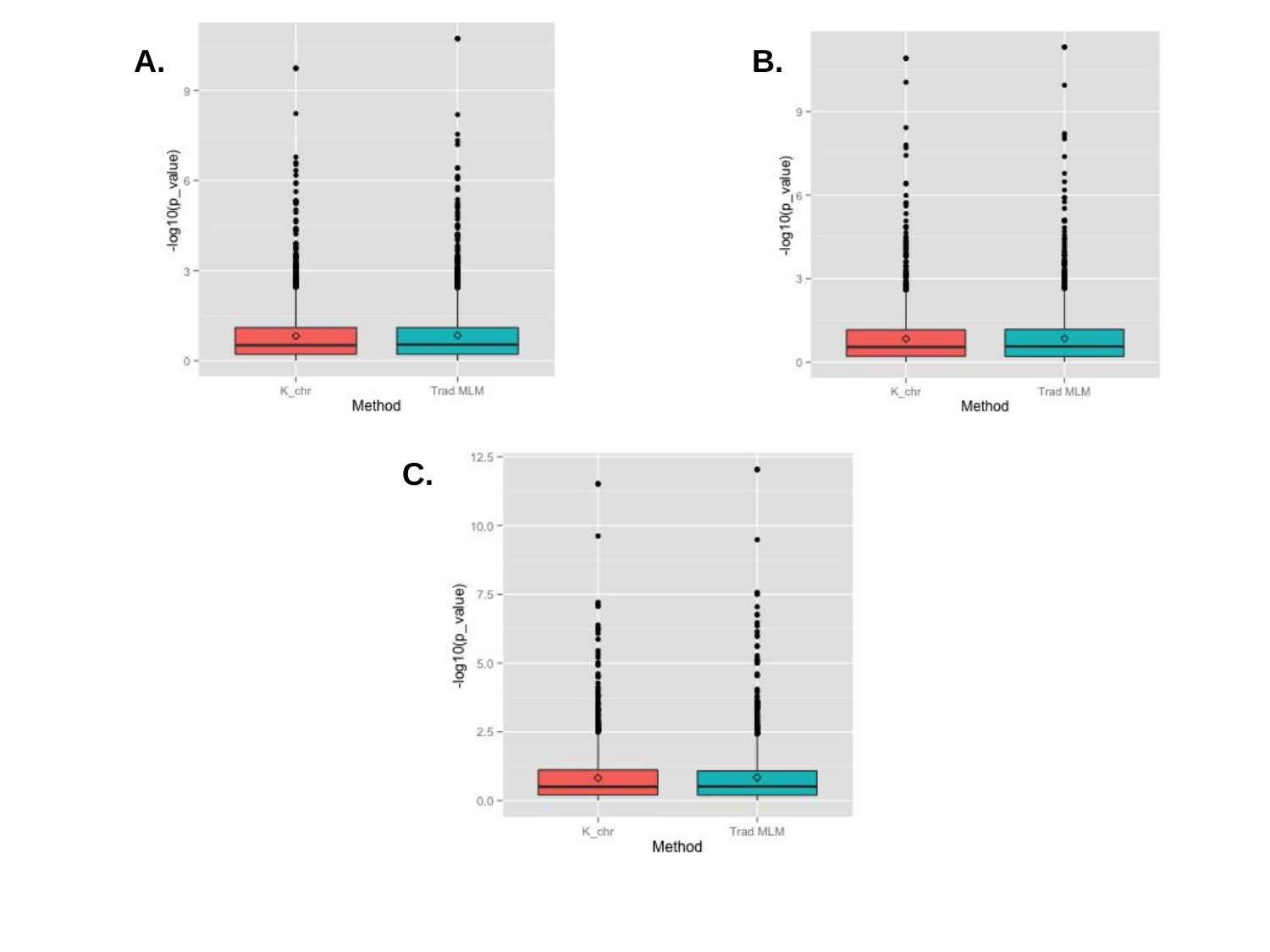

A.
B.
C.

Supplement: Supplemental Material [file supp_g3.116.029090_FigureS4.pptx]

## Slide 1
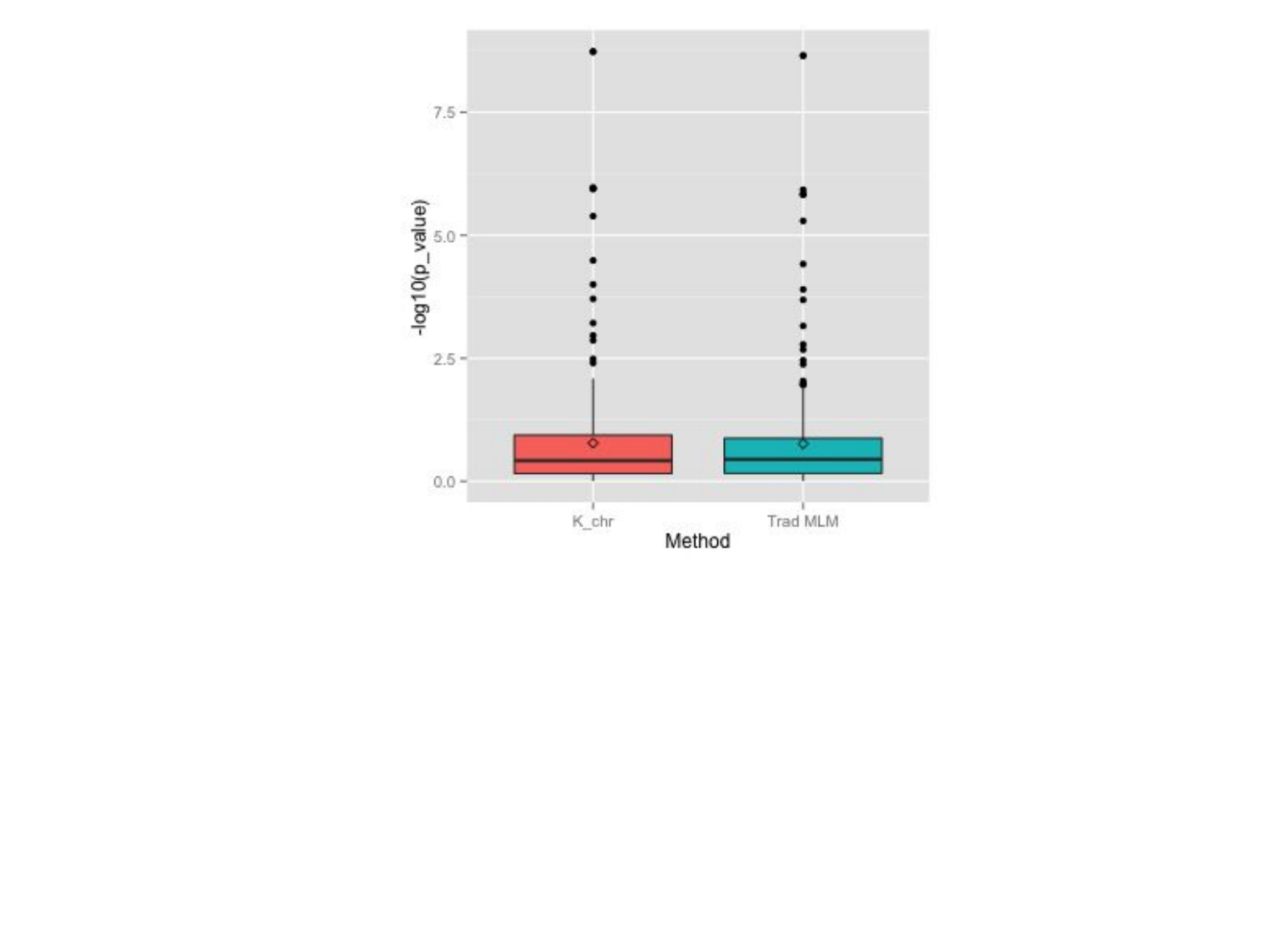

Supplement: Supplemental Material [file supp_g3.116.029090_FigureS5.pptx]

## Slide 1
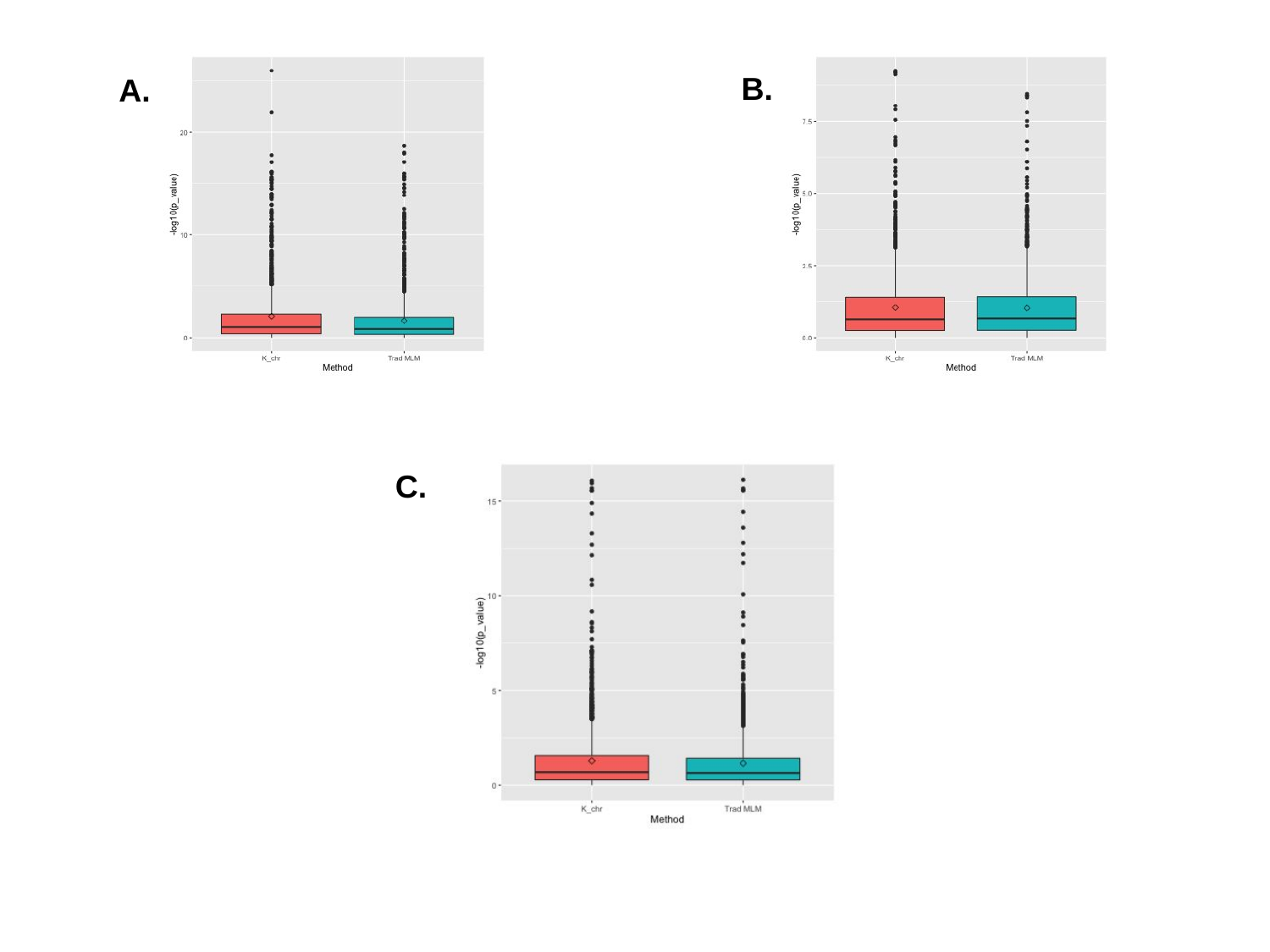

B.
A.
C.

Supplement: Supplemental Material [file supp_g3.116.029090_FigureS6.pptx]
